# (Bad) Feelings about Meeting Them? Episodic and Chronic Intergroup Emotions Associated with Positive and Negative Intergroup Contact As Predictors of Intergroup Behavior

**DOI:** 10.3389/fpsyg.2017.01449

**Published:** 2017-08-29

**Authors:** Mathias Kauff, Frank Asbrock, Ulrich Wagner, Thomas F. Pettigrew, Miles Hewstone, Sarina J. Schäfer, Oliver Christ

**Affiliations:** ^1^Institute of Psychology, FernUniversität in Hagen Hagen, Germany; ^2^Department of Psychology, Technische Universität Chemnitz Chemnitz, Germany; ^3^Faculty of Psychology, Philipps-Universität Marburg Marburg, Germany; ^4^Department of Psychology, University of California, Santa Cruz, Santa Cruz CA, United States; ^5^Oxford Centre for the Study of Intergroup Conflict, University of Oxford Oxford, United Kingdom

**Keywords:** intergroup contact, intergroup emotions, intergroup behavior

## Abstract

Based on two cross-sectional probability samples (Study 1: *N* = 1,382, Study 2: *N* = 1,587), we studied the interplay between positive and negative intergroup contact, different types of intergroup emotions (i.e., episodic intergroup emotions encountered during contact and more general chronic intergroup emotions), and outgroup behavior in the context of intergroup relations between non-immigrant Germans and foreigners living in Germany. In Study 1, we showed that positive and negative contact are related to specific episodic intergroup emotions (i.e., anger, fear and happiness). Results of Study 2 indicate an indirect effect of episodic intergroup emotions encountered during contact experiences on specific behavioral tendencies directed at outgroup members via more chronic situation-independent intergroup emotions. As expected, anger predicted approaching (discriminatory) behavioral tendencies (i.e., aggression) while fear predicted avoidance. The results extend the existing literature on intergroup contact and emotions by addressing positive and negative contact simultaneously and differentiating between situation-specific episodic and chronic intergroup emotions in predicting discriminatory behavioral tendencies.

## Introduction

**FIGURE 1:**
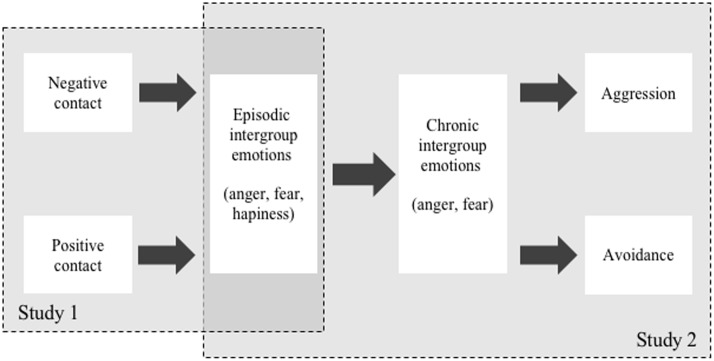
Theoretical model to be tested in Studies 1 and 2.

Intergroup contact theory can be considered as one of the most well-researched social-psychological theories dealing with intergroup relations and the reduction of outgroup prejudice (e.g., Pettigrew and Tropp, 2011). The majority of studies in the field of intergroup contact focus on consequences of positive contact experiences. Although research on the effects of negative intergroup contact has recently increased, it is still rather scarce compared to studies addressing positive contact (but see for example Pettigrew and Tropp, 2006; Graf et al., 2014; Hayward et al., 2017; Reimer et al., 2017; for an overview see Pettigrew and Hewstone, 2017). Likewise, the role of specific emotions associated with intergroup contact experiences in explaining intergroup relations has only seldom been studied (Paolini et al., 2006; Techakesari et al., 2015; Seger et al., 2016; Visintin et al., 2017) – especially in the context of negative contact experiences (Hayward et al., 2017). Nor have intergroup emotions related to positive and negative intergroup contact been used to predict specific behavioral tendencies directed at outgroups (however, there is a substantial set of studies on the mediating role of anxiety in the contact-attitudes link, for an overview see Paolini et al., 2015). Within the present study, we analyze the relationship between positive as well as negative contact, different emotions, and discriminatory behavioral tendencies targeted at outgroups, while additionally differentiating between aggression and avoidance. We specifically sought to test (a) whether positive and negative contact experiences are related to different transient episodic emotions encountered during the contact experience (i.e., anger, fear, and happiness; Study 1), (b) how these episodic emotions are related to more chronic intergroup emotions (Paolini et al., 2006), and (c) how the latter are related to specific forms of discriminatory behavioral tendencies (i.e., aggression and avoidance; Study 2; for an overview see **Figure 1**).

## Positive and Negative Intergroup Contact

Following Allport’s proposal that contact between members of different groups helps to overcome mutual enmity (Allport, 1954), scholars have presented a plethora of empirical evidence that (positive) intergroup contact reduces prejudice (e.g., Hewstone, 2009; Pettigrew and Tropp, 2011). The majority of research to date, however, has focused on the outcomes of positive intergroup contact. In their extensive meta-analysis, Pettigrew and Tropp (2006, p. 767; see also Dixon et al., 2005) have pointed out that “past contact research is limited by its primary emphasis on positive features of the contact situation.” Positive and negative intergroup contact have seldom been explicitly defined. Most researchers, however, refer to positive contact when contact is positively evaluated because it is accompanied by beneficial outcomes for an interaction partner (e.g., cooperative interactions with positive joint outcomes, learning, or feelings of appreciation). Negative contact, on the other hand, is characterized by interactions that are negatively evaluated because of negative outcomes (e.g., competitive interactions with negative outcomes for one of the interaction partners, hostile behavior by the interaction partner, or feelings of being exploited; e.g., Hayward et al., 2017). While it is obvious that positive contact with outgroup members has a positive influence on outgroup attitudes and behavior, it has long been unclear how negative contact relates to outgroup attitudes and behavior. Recent studies comparing positive and negative contact indicate that negative contact is less common than positive contact (Pettigrew and Tropp, 2011; Graf et al., 2014) but seems to have stronger effects on prejudice (i.e., increasing negative attitudes) than positive contact (Barlow et al., 2012; Graf et al., 2014). Moreover, some researchers have started to look at interaction effects of positive and negative contact experiences on outgroup attitudes (Pettigrew and Tropp, 2011; Techakesari et al., 2015; Fell et al., unpublished) and collective action (Reimer et al., 2017). Beside these studies, however, negative contact effects – in addition to positive contact – have seldom been taken into account as predictors of intergroup attitudes and behavior.

Likewise, although mediators of the prejudice-reducing effect of positive contact have been studied extensively (e.g., Pettigrew and Tropp, 2008), not much is known about the processes underlying the relationship between negative intergroup contact and negative outgroup attitudes and, more importantly, behavior. We propose that intergroup emotions are worth considering in this regard (cf. Seger et al., 2016).

We turn now to the importance of studying the role of intergroup emotions in the field of intergroup relations in general and intergroup contact specifically. Building on a taxonomy of different types of emotions (i.e., chronic and episodic emotions), we will then detail the role of emotions in our theoretical framework and the aims of the present study.

## Intergroup Emotions

As Smith and Mackie (2005) pointed out, the successful years of intergroup research have been characterized by a strong dominance of a social cognitive orientation. Emotions related to intergroup phenomena were widely ignored. However, lately different scholars have highlighted that emotions experienced on behalf of a social group (i.e., intergroup emotions) predict behavior toward outgroup members over and above cognitive evaluations of outgroups (e.g., van Zomeren et al., 2008). Talaska et al. (2008), for example, demonstrated in a meta-analysis of 57 studies that what they call emotional prejudice (i.e., feelings toward outgroups, that is, intergroup emotions) is a much stronger predictor of observed and self-reported discrimination than are cognitive aspects of prejudice (i.e., stereotypic beliefs about the outgroup). Moreover, Stangor et al. (1991) showed that affective responses to national, ethnic, and religious groups were a more consistent predictor of attitudes and social distance than stereotypic beliefs about these groups. Cuddy et al. (2007) demonstrated across four studies that intergroup emotions such as admiration, envy, contempt, and pity predicted helpful and harmful behavioral tendencies better than cognitive stereotypes (see also Cottrell and Neuberg, 2005).

### Contact and Intergroup Emotions

Following up on these findings, Mackie and Smith (2015) argued that a substantial understanding of intergroup processes needs to consider intergroup emotions in addition to an analysis of social cognition. In line with this notion, some researchers studied the role of emotions for the relationship between contact and outgroup attitudes and behavior. Tropp and Pettigrew (2005), for example, showed that intergroup contact especially reduces affective components of prejudice. Moreover, it is widely acknowledged that anxiety is a key mediator in the positive contact-attitudes link (Aberson and Haag, 2007; Aberson and McVean, 2008; Pettigrew and Tropp, 2008; Tausch et al., 2009; Swart et al., 2011). However, only a few studies have looked at additional intergroup emotions. In one rare example, Miller et al. (2004) examined the effects of intergroup contact on prejudice, mediated by intergroup emotions. In two cross-sectional questionnaire studies, they showed that the effects of past intergroup contact on prejudice were mediated by emotions experienced during intergroup contact – even when controlling for cognitive measures of stereotypes. Positive intergroup contact reduced negative (e.g., angry or afraid) and enhanced positive (e.g., respectful or sympathetic) emotions toward the outgroup (see also Visintin et al., 2017). In addition, Seger et al. (2016) differentiated between specific positive and negative intergroup emotions and showed that admiration, anger, and disgust are important mediators in the link between positive intergroup contact and outgroup attitudes. Neither Miller et al. (2004) nor Seger et al. (2016), however, distinguished between different types of emotions (i.e., episodic, situation-specific emotions and more general chronic emotions). In a recent study, Kenworthy et al. (2015) analyzed the interplay of intergroup contact and intergroup emotions in Northern Ireland. They showed that cross-group friendships predicted positive and negative intergroup emotions, which in turn were associated with distinct behavioral tendencies. Kenworthy and colleagues differentiated between specific positive and negative chronic emotions. However, they did not study negative forms of intergroup contact. In fact, only a few studies have addressed intergroup emotions as mediators of negative contact effects. Techakesari et al. (2015) showed that across various intergroup contexts positive and negative contact experiences influence feelings of intergroup anxiety, which, in turn, predict prejudice and negative metaperceptions. Moreover, Mazziotta et al. (2015) demonstrated a mediating effect of intergroup anxiety for the relationship between positive as well as negative extended contact (i.e., knowing that other ingroup members have contact) and direct contact. However, none of these studies addressed the relationship between positive as well as negative contact and intergroup emotions beyond intergroup anxiety. In one recent and rare example, Hayward et al. (2017) showed that anxiety and anger are relevant mediators for both positive and negative contact, whereas empathy is especially relevant to understand positive contact effects. Our research adds to this study by additionally investigating the role of happiness. We consider this important because prior research illustrates the importance of positive intergroup emotions (e.g., Fiske et al., 2002; Cuddy et al., 2007). Moreover, happiness has been shown to predict approach-behavior toward groups (Kessler and Hollbach, 2005).

Most research in the field of intergroup emotions has focused implicitly on generalized and chronic emotions (but see for example Miller et al., 2004). In the present study, however, we differentiate between such *chronic* intergroup emotions and *episodic* emotions encountered within the contact situations. This differentiation is guided by a taxonomy of different types of emotions introduced by Paolini et al. (2006). In their review, Paolini et al. (2006) systematically discussed the role of intergroup emotions for intergroup contact effects. They differentiate between two types of non-incidental emotions, namely episodic and chronic intergroup emotions. While episodic emotions are transient, targeted at specific outgroup members, and dependent on specific intergroup situations, chronic emotions involve “enduring and stable affective reactions to social groups and their members” (Paolini et al., 2006, p. 215). Despite being situation specific and transient, episodic emotions are considered as intergroup emotions because they are experienced in an intergroup context and reflect reactions toward an outgroup member (see also Iyer and Leach, 2008). Chronic intergroup emotions are defined as affective components of negative attitudes toward outgroups (e.g., Zanna and Rempel, 1988). As such, Paolini et al. (2006) argued that chronic intergroup emotions are influenced by episodic affective experiences In other words, repeatedly experienced emotions in specific intergroup situations can lead to more general and chronic intergroup emotions. In line with this notion, Aberson (2015) showed that Whites’ positive and negative feelings during interactions with African Americans were both related to more positive and negative affect toward African Americans in general.

Paolini et al. (2006) not only argued that episodic intergroup emotions influence chronic intergroup emotions but, additionally, that chronic intergroup emotions as affective attitudes predict intergroup behavior. However, to our knowledge this full theoretical model has not yet been tested. Across two studies, we studied the validity of different parts of the model.

## The Present Study

The goal of the present research was to study how positive and, more importantly, negative intergroup contact relates to situation-specific episodic emotions and how these episodic emotions are associated with affective outgroup attitudes, that is chronic intergroup emotions. Moreover, we tested the relationship of such chronic intergroup emotions and specific behavioral tendencies (i.e., approach versus avoidance) directed at outgroup members.

Hence, we sought to address two research gaps in our research. First, we focused on situation-specific episodic intergroup emotions as consequences of both positive and negative intergroup contact experiences. Second, we analyzed the relationship between episodic and chronic intergroup emotions and used the unique predictive power of affective intergroup attitudes (i.e., chronic intergroup emotions) to predict specific discriminatory behavioral tendencies.

In Study 1, which was partly exploratory, we analyzed the association between positive and negative contact and three different episodic emotions encountered during contact, namely anger, fear, and happiness. In Study 2, we again measured episodic emotions encountered during contact, but additionally introduced chronic intergroup emotions (anger and fear) as well as discriminatory behavioral tendencies directed at the outgroup (active and passive discrimination). Based on the idea that intergroup emotions lead to specific forms of actions (e.g., Cottrell and Neuberg, 2005; Smith and Mackie, 2005; Paolini et al., 2006; Cuddy et al., 2007) we hypothesize that chronic anger is related to active forms of discriminatory behavior (i.e., aggression; see Mackie et al., 2000) while chronic fear relates to passive forms of discriminatory behavior (i.e., avoidance; see also Wagner and Christ, 2007).

## Study 1

In Study 1, we analyzed the relationship between positive and negative contact and episodic emotions experienced in intergroup contact. In line with Seger et al. (2016), we assume that positive contact is associated with an increase in positive and a decrease in negative episodic emotions. Negative contact, on the other hand, should be related to more negative and less positive episodic emotions (see also Hayward et al., 2017).

### Method

Data of Study 1 (*N* = 1,383) stem from a cross-sectional German probability telephone survey of the autochthonous German adult (16 years of age and older) population (for details see Heitmeyer, 2005).^1^ The fieldwork took place during the summer of 2004. Since data were derived from a contracted survey company the strict ethics code of the survey company (name of research company: Infratest Sozialforschung, München) was followed. Therefore, no formal ethics approval was requested by a university ethics committee. Informed consent was provided orally since that was the only practical means of doing so without compromising people’s anonymity. Respondents were assured that participation was voluntary, could be stopped at any time and that data were anonymized. No identifying information such as name, address or telephone number was collected. Data were anonymous when handed over to researchers.

Study 1 included two indicators each for *negative* (‘How often has a foreigner bothered you?’, ‘How often has a foreigner intimidated you?’), and *positive* (‘How often has a foreigner helped you?’, ‘How often have you had an interesting conversation with a foreigner?’) contact experiences. The intercorrelations within the indicators were satisfactory for both negative (*r* = 0.59; *p* < 0.001) and positive (*r* = 0.54; *p* = 0.001) contact. Higher values indicate more negative and more positive contact experiences, respectively. Measures of three forms of *episodic intergroup emotions* were included in the survey. Respondents were instructed to think of situations in which they had contact with foreigners in Germany and indicate how often they had felt one of the listed emotions. The indicators focused on episodic intergroup emotions experienced in the contact situation; that is, items were introduced with the sentence: “Thinking about contact situations with foreigners living in Germany, how often did you encounter the following emotions?”. Each emotion was tapped with two items: anger (‘angry’, ‘irritated’; *r* = 0.69; *p* < 0.001), fear (‘frightened,’ ‘helpless’; *r* = 0.50; *p* < 0.001); and happiness (‘happy,’ ‘satisfied’; *r* = 0.76; *p* < 0.001). Higher values indicate more negative and more positive emotions, respectively. In the following, we refer to these forms of intergroup emotions as episodic fear, episodic anger, and episodic happiness. All indicators were answered on a four-point response scale (*1* = *never* to *4* = *very often*). We emphasize that indicators for both intergroup contact and episodic intergroup emotions do not focus on specific instances or situations. Rather, they represent measures of aggregated contact experiences and emotions encountered within these situations.

### Results and Discussion

All analyses are based on covariance matrices and robust maximum likelihood estimation (Mplus 7.4, Muthén and Muthén, 1998–2015). Furthermore, the hierarchical structure of the data was taken into account using the ‘complex’ procedure implemented in Mplus (Muthén and Satorra, 1995).^2^ Full information maximum likelihood was used to handle missing data (Schafer and Graham, 2002; missing values in no case exceeded more than three percent). Model comparison using the Chi-square difference test is based on corrected Chi-square values (Satorra and Bentler, 2001). **Table 1** lists the descriptive statistics as well as intercorrelations of the measures of Study 1.

**Table 1:** Means, standard deviations, and intercorrelations of measures for Study 1.

	*M*	*SD*	2	3	4	5
1 Positive contact	2.09	0.73	0.01	-0.05	-0.14^∗∗∗^	0.53^∗∗∗^
2 Negative contact	1.36	0.56		0.54^∗∗∗^	0.40^∗∗∗^	-0.13^∗∗∗^
3 Episodic anger	1.59	0.66			0.49^∗∗∗^	-0.19^∗∗∗^
4 Episodic fear	1.38	0.56				-0.21^∗∗∗^
5 episodic happiness	2.56	0.75				

^∗∗∗^*p < 0.001*.

Positive and negative contact were operationalized with items reflecting examples of positive and negative experiences, rather than broader evaluations of the valence of contact. Hence, they do not comprise an emotional evaluation of the contact situations. We assumed that, and tested whether, intergroup contact and episodic intergroup emotions represent independent constructs. Hence, before testing the overall model, we ran confirmatory factor analyses (CFA) to analyze whether positive/negative contact and positive/negative episodic intergroup emotions could be separated, or if they formed two correlated factors (positive vs. negative intergroup contact). The analyses clearly showed that a model with five correlated factors separating positive and negative contact and episodic intergroup emotions (χ^2^ = 65.13, df = 25, *p* < 0.001, CFI = 0.988, RMSEA = 0.034, SRMR = 0.021) fits the data satisfactorily and significantly better than the alternative two-factor model combining correspondent valence of contact and episodic intergroup emotions (χ^2^ = 560.01, df = 34, *p* < 0.001, CFI = 0.843, RMSEA = 0.106, SRMR = 0.064; Δχ^2^ = 494.88; df = 9; *p* < 0.001).^3^

**FIGURE 2:**
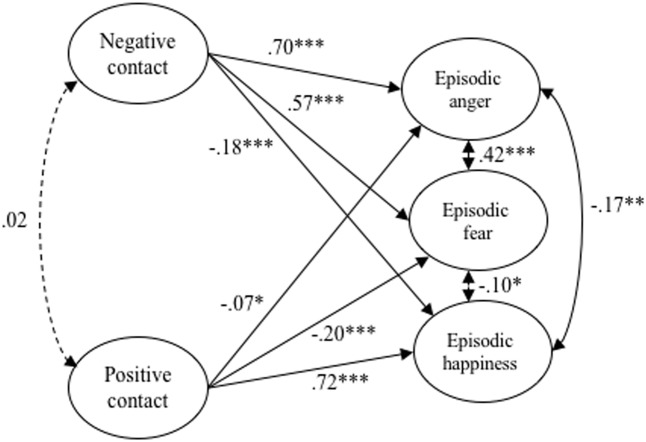
Structural equation model tested in Study 2. ^∗∗∗^*p* < 0.001, ^∗∗^*p* < 0.01, ^∗^*p* < 0.05; standardized coefficients are shown. Continuous arrows indicate significant, and dashed arrows non-significant, relationships. Double-headed arrows indicate correlations. Indicator variables of latent variables and error variances are not included for simplicity; (χ^2^ = 65.13; df = 25; *p* < 0.001; CFI = 0.988; RMSEA = 0.034; SRMR = 0.021).

We then estimated a structural equation model in which episodic intergroup emotions are regressed on positive and negative contact. The structural model showed an acceptable fit (χ^2^ = 69.13, df = 25, *p* < 0.001, CFI = 0.988, RMSEA = 0.034, SRMR = 0.021; see **Figure 2** for an overview of the model including standardized coefficients). Negative contact experiences were reliably associated with all episodic intergroup emotions, as expected positively associated with anger (*b* = 0.84, *SE* = 0.059, *p* < 0.001, *CI_95%_* = 0.729, 0.959) and fear (*b* = 0.61, *SE* = 0.053, *p* < 0.001, *CI_95%_* = 0.503, 0.713), and negatively associated with happiness (*b* = -0.24, *SE* = 0.042, *p* < 0.001, *CI_95%_* = -0.318, -0.154). Likewise, positive contact experiences were reliably associated with all episodic intergroup emotions; again as expected, negatively with anger (*b* = -0.08, *SE* = 0.035, *p* = 0.03, *CI_95%_* = -0.147, -0.007) and fear (*b* = -0.20, *SE* = 0.040, *p* < 0.001, *CI_95%_* = -0.281, -0.125), and positively with happiness (*b* = 0.91, *SE* = 0.060, *p* < 0.001, *CI_95%_* = 0.796, 1.030).

The present results indicate that both positive and negative contact experiences are associated with positive and negative episodic intergroup emotions. Interestingly, and in contrast to a recent study by Hayward et al. (2017), positive contact was more strongly associated with positive emotions than with negative emotions, and negative contact was more strongly associated with negative emotions than with positive emotions (i.e., the respective absolute values of the 95% confidence intervals did not overlap).

Moreover, the incidence rates show that it is extremely important to take the mean differences of the different forms of intergroup contact experiences into account. The two positive emotions (happiness and satisfaction in the contact situation) were both experienced by 91% of the respondents at least occasionally, while for the negative emotions the percentages were only 56% (angry), 48% (irritated), 34% (anxious) and 25% (helplessness). Thus positive emotional contact experiences occur far more often and could hence be more formative in everyday life than negative contact experiences (Graf et al., 2014; Hayward et al., 2017).

To summarize, Study 1 should be considered as a first step: Results support the idea that intergroup contact is associated with episodic emotions. In Study 2, we sought to test a more complex model and studied the relationship between episodic emotions encountered during contact, more chronic emotions, and specific discriminatory behavioral intentions directed at outgroup members.

## Study 2

In Study 2, we sought to extend the focus of Study 1 by studying the relationship between specific episodic and chronic intergroup emotions as well as two different types of discriminatory behavioral tendencies, namely aggressive behavior toward outgroup members and avoidance of outgroup members. We tested whether an indirect effect of episodic emotions experienced during intergroup contact on aggressive and avoidant behavioral tendencies via more general and chronic intergroup emotions occurred. Moreover, in line with the recent literature on intergroup emotions, we hypothesize that episodic and chronic anger are primarily related to approach behavior (i.e., aggression; Mackie et al., 2000; Cottrell and Neuberg, 2005) while episodic and chronic fear are related to avoidance behavior (Mackie et al., 2009).

### Method

For Study 2, we used data from a different German probability telephone survey (*N* = 1,778) of the German adult (16 years of age and older) population from the same project (for details see Heitmeyer, 2006, for information about the project as well as ethical approval see Study 1). The field phase of this survey occurred during the summer of 2005. For analyses in Study 2, only those respondents (*N* = 1,587) were used who indicated that they had any contact with foreigners in Germany because in the survey respondents who indicated having no contact were excluded from answering other relevant questions. The survey contained measures of episodic emotions encountered during contact, but no measures of positive or negative intergroup contact experiences.

Measures of the three forms of *episodic intergroup emotions* (episodic anger, episodic fear, and episodic happiness during intergroup contact situations with foreigners) were the same as in Study 1. *Chronic intergroup emotions* were measured with one item for anger (‘Turks in Germany sometimes make me angry’), and one for fear (‘Turks in Germany make me afraid’). Both indicators focus on emotions related to the outgroup in general. Higher values indicate stronger emotions. Two forms of *discriminatory behavioral tendencies* were assessed with two items each. The indicators for tendencies to behave aggressively against the outgroup were: ‘If a Turk insults me, I might hit him’ and ‘If really necessary, I’m ready to make Turks respect me by using violence’ (*r* = 0.48; *p* < 0.001). Two further indicators measured the tendency to avoid the outgroup: ‘If possible I avoid sitting next to a Turk on a bus’ and ‘If a Turk speaks to me I’m reserved’ (*r* = 0.59; *p* < 0.001). Higher values indicate stronger action tendencies. All indicators were answered on a four-point response scale (*1* = *never* to *4* = *very often* and *1* = *full disagreement* to *4* = *full agreement*).

It might first appear that the episodic intergroup emotion indicators, the chronic intergroup emotions measures, and the behavioral tendency measures do not correspond exactly, since the episodic intergroup emotion items target foreigners in general while the other measures target Turks. However, in Germany these two target groups are widely considered as identical. Turkish migrants are not only the largest immigrant group (Federal Agency for Civic Education, 2015) but also constitute the prototype of foreigners living in Germany (Asbrock et al., 2014).

### Results and Discussion

**Table 2** summarizes the means and intercorrelations of measures of Study 2.

**Table 2:** Means, standard deviations, and intercorrelations of measures for Study 2.

	Study 2							
	*M*	*SD*	2	3	4	5	6	7
1 Episodic anger	1.66	0.68	0.38^∗∗∗^	-0.29^∗∗∗^	0.46^∗∗∗^	0.31^∗∗∗^	0.26^∗∗∗^	0.23^∗∗∗^
2 Episodic fear	1.36	0.50		-0.13^∗∗∗^	0.26^∗∗∗^	0.34^∗∗∗^	0.15^∗∗∗^	0.21^∗∗∗^
3 Episodic happiness	2.54	0.67			-0.23^∗∗∗^	-0.19^∗∗∗^	-0.18^∗∗∗^	-0.24^∗∗∗^
4 Chronic anger	2.00	0.87				0.58^∗∗∗^	0.32^∗∗∗^	0.44^∗∗∗^
5 Chronic fear	1.77	0.74					0.22^∗∗∗^	0.44^∗∗∗^
6 Aggression	1.59	0.72						0.30^∗∗∗^
7 Avoidance	1.73	0.68						

^∗∗∗^*p < 0.001*.

We tested if the two types of emotions in the intergroup context – episodic and chronic emotions – could be separated, by comparing CFA results for three models: (a) all emotion indicators are restricted to load on one latent factor; (b) indicators of emotions are separated into three different correlated factors – anger, fear, and happiness; and (c) episodic emotions are separated from chronic intergroup emotions resulting in five correlated factors. Fit for model (a) was poor (χ^2^ = 960.92; df = 20; *p* < 0.001; CFI = 0.622; RMSEA = 0.172; SRMR = 0.098) showing that a one factorial solution does not represent the observed covariance matrix. Differentiating the various emotions without separating episodic and chronic intergroup emotions in model (b) also resulted in a poor model fit (χ^2^ = 377.85; df = 17; *p* < 0.001; CFI = 0.855; RMSEA = 0.116; SRMR = 0.059), although it was significantly better than the one factorial solution (Δχ^2^ = 512.58; df = 3; *p* < 0.001). Model (c), differentiating five different types of emotions, fitted the data satisfactorily (χ^2^ = 20.85; df = 12; *p* = 0.05; CFI = 0.996; RMSEA = 0.022; SRMR = 0.013) and significantly better than model (b) (Δχ^2^ = 347.07; df = 5; *p* < 0.001).^4^ Hence, the CFAs convincingly supported our proposition that two general types of emotions in the intergroup context – episodic and chronic intergroup emotions – can be differentiated. In addition, these general types can be further differentiated into distinct emotions.

**FIGURE 3:**
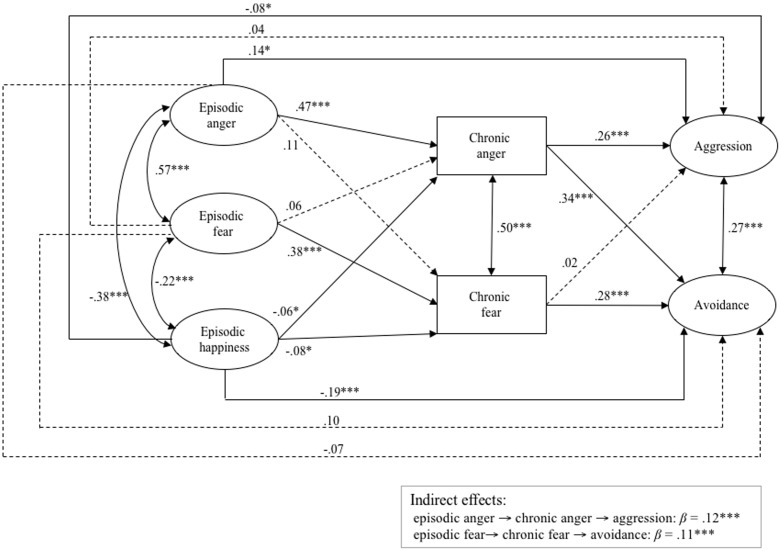
Structural equation model tested in Study 3. ^∗∗∗^*p* < 0.001, ^∗^*p* < 0.05; standardized coefficients are shown. Continuous arrows indicate significant, and dashed arrows non-significant, relationships. Double-headed arrows indicate correlations. Indicator variables of latent variables and error variances are not included for simplicity; (χ^2^ = 52.98; df = 35; *p* < 0.001; CFI = 0.995; RMSEA = 0.018; SRMR = 0.015).

We then estimated a structural equation model in which chronic intergroup emotions were regressed on episodic emotions encountered during contact, and discriminatory intentions were regressed on chronic intergroup emotions. Discriminatory intentions were additionally regressed on episodic intergroup emotions to test whether indirect effects of episodic emotions on discriminatory tendencies via chronic intergroup emotions occurred. In line with our hypothesis that fear should relate to avoidance action tendencies, and anger to approach action tendencies, we estimated the direct and indirect effect of episodic anger (via chronic anger) on aggression, and episodic fear (via chronic fear) on avoidance. The structural model showed a good fit (χ^2^ = 52.98, df = 35, *p* < 0.001, CFI = 0.995, RMSEA = 0.018, SRMR = 0.015; see **Figure 3** for an overview of the model including standardized coefficients).

Episodic anger (*b* = 0.66, *SE* = 0.071, *p* < 0.001, *CI_95%_* = 0.523, 0.803) and episodic happiness (*b* = -0.11, *SE* = 0.048, *p* = 0.03, *CI_95%_* = -0.201, -0.014), but not episodic fear (*b* = 0.12, *SE* = 0.093, *p* = 0.20, *CI_95%_* = -0.063, 0.300), were associated with chronic anger. Episodic fear (*b* = 0.65, *SE* = 0.114, *p* < 0.001, *CI_95%_* = 0.424, 0.872) and episodic happiness (*b* = -0.12, *SE* = 0.046, *p* = 0.02, *CI_95%_* = -0.208, -0.027), but not episodic anger (*b* = 0.13, *SE* = 0.068, *p* = 0.07, *CI_95%_* = -0.008, 0.260), were associated with chronic fear. Moreover, chronic anger was associated with aggression (*b* = 0.21, *SE* = 0.041, *p* < 0.001, *CI_95%_* = 0.126, 0.288) and avoidance (*b* = 0.22, *SE* = 0.028, *p* < 0.001, *CI_95%_* = 0.165, 0.276), while chronic fear was associated with avoidance (*b* = 0.22, *SE* = 0.038, *p* < 0.001, *CI_95%_* = 0.142, 0.290), but not aggression (*b* = 0.02, *SE* = 0.043, *p* = 0.74, *CI_95%_* = -0.072, 0.099). Episodic anger (*b* = 0.16, *SE* = 0.069, *p* = 0.03, *CI_95%_* = 0.020, 0.291) and episodic happiness (*b* = -0.11, *SE* = 0.050, *p* = 0.03, *CI_95%_* = -0.205, -0.010), but not episodic fear (*b* = 0.06, *SE* = 0.098, *p* = 0.55, *CI_95%_* = -0.133, 0.251), were directly associated with aggression. Only episodic happiness (*b* = -0.21, *SE* = 0.037, *p* < 0.001, *CI_95%_* = -0.283, -0.137), but neither episodic anger (*b* = -0.06, *SE* = 0.061, *p* = 0.32, *CI_95%_* = -0.060, 0.059) nor episodic fear (*b* = 0.13, *SE* = 0.093, *p* = 0.17, *CI_95%_* = -0.054, 0.312), were directly associated with avoidance. Most importantly, we found an indirect effect of episodic anger on aggression via chronic anger (specific indirect effect *b* = 0.14, *SE* = 0.032, *p* < 0.001, *CI_95%_* = 0.074, 0.200), as well as an indirect effect of episodic fear on avoidance via chronic fear (specific indirect effect *b* = 0.14, *SE* = 0.032, *p* < 0.001, *CI_95%_* = 0.077, 0.203).^5^

These results support our assumption that positive and negative episodic emotions experienced during intergroup contact relate to discriminatory behavioral tendencies, and that there are indirect effects of episodic intergroup emotions on discriminatory behavioral tendencies via chronic intergroup emotions. As expected, the total effect of the association between episodic anger and aggressive tendencies was significant and positive, as was the total effect of the association between episodic fear and avoidant tendencies. Moreover, the direct effect of the association between episodic happiness and both discriminatory tendencies was significant and negative.

Results of Study 2 confirmed our proposition that episodic and chronic emotions are distinct but related constructs: episodic fear and anger during contact (and therefore arising from or directed at the outgroup member(s) present in the contact situation) had specific effects on their counterparts on the level of chronic intergroup emotions (directed at the outgroup in general). Moreover, chronic intergroup emotions were directly associated with both approach (i.e., aggression) and avoidance action tendencies. Chronic anger was directly associated with aggressive and, unexpectedly, with outgroup avoiding tendencies (see also Wagner and Christ, 2007; Hayward et al., 2017). A plausible explanation for this unexpected path would be that people who are angry about the outgroup try to deal with this feeling by avoiding members of the outgroup. Chronic fear, however, as assumed, was only directly associated with avoidance behavior tendencies. This particular finding is in line with research showing that individuals experiencing chronic fear of outgroups (i.e., authoritarians) typically avoid outgroup members (Pettigrew and Tropp, 2011). Furthermore, we found evidence for indirect effects of episodic emotions encountered during contact on action tendencies toward the outgroup via chronic intergroup emotions. It is noteworthy that we were unable to measure a chronic counterpart for episodic happiness. However, episodic happiness showed small but significant associations with chronic anger and fear as well as with active and passive behavioral tendencies.

## General Discussion

In the present research, we analyzed two types of intergroup emotions as consequences of contact experiences as well as predictors of outgroup behavior. In doing so, our results extend previous research on intergroup contact and intergroup emotions (e.g., Kenworthy et al., 2015). We showed in two large probability surveys, that positive and negative contact, on the one hand, and episodic and chronic intergroup emotions, on the other hand, are distinct but related constructs. More precisely, we demonstrated that (a) positive *and* negative intergroup contact experiences are related to episodic intergroup emotions of fear, anger, and happiness and (b) that these episodic intergroup emotions are associated with chronic intergroup emotions which again are predictors of specific behavioral tendencies targeted at outgroup members. Furthermore, our results indicate an indirect relationship between episodic emotions and aggressive and avoidant behavioral tendencies toward outgroup members via chronic intergroup emotions. It is important to note, however, that Study 2 showed a remaining direct effect from episodic emotions during contact to action tendencies, even after controlling for chronic intergroup emotions.

Our results are in line with Paolini et al.’s (2006) notion that chronic intergroup emotions represent the affective component of attitudes and hence predict behavioral components of attitudes, that is, specific behavioral tendencies (Smith, 1993; see also Cottrell and Neuberg, 2005; Smith and Mackie, 2005; Cuddy et al., 2007). In supporting this idea of a link between chronic intergroup emotions and specific behavior the present research also replicates and extends Aberson’s (2015) study, which showed that positive and negative contact are associated with positive and negative emotions during that contact experience, and these emotions predict chronic affective attitudes. Aberson, however, did not study specific behavioral tendencies (and used less specific indicators of intergroup emotions; see also Parkinson et al., 2005; Hayward et al., 2017).

It is important to note, however, that although our research demonstrates the importance of emotions in intergroup relations, one can also argue that additional processes besides intergroup emotions are also relevant - such as cognitive processes of stereotype change (e.g., Hewstone, 1994; Wolsko et al., 2003) and processes of recategorization (e.g., Brewer and Miller, 1984; Gaertner and Dovidio, 2000).

Notwithstanding the contributions of our research, we acknowledge two major limitations of our studies. First, the studies are correlational – implying the usual shortcoming in establishing causal links between the variables analyzed. Therefore, our work should be regarded as a starting point, and future research should incorporate experimental and longitudinal studies to get a more accurate picture of the causal influences between the constructs involved.

Second, our analyses are based on secondary data. Therefore, some of the measures we made use of are suboptimal. Study 2, for example, lacks not only differentiated measures of positive and negative intergroup contact, but also an equivalent measure for episodic happiness on the level of chronic intergroup emotions. Furthermore, the items tapping episodic emotions demand an aggregated retrospective assessment of emotions encountered during contact. Hence, when referring to Paolini et al.’s (2006) taxonomy they can only be considered as a proxy for episodic emotions. In addition, chronic intergroup emotions of anger and fear were measured with a single item only. Furthermore, we cannot rule out that the different outgroup targets used (foreigners vs. Turks) and response scaling of measures for episodic and chronic intergroup emotions in Study 2 allowed for a separation of the respective constructs in confirmatory factor analyses. Moreover, across both studies, the realm of emotions analyzed was necessarily restricted in such large-scale telephone surveys (albeit still broader than in previous studies on emotional consequences of positive and negative contact). We could only analyze the effects of three emotions, two negative and one positive. Other researchers have described various other important emotions in the intergroup context, such as envy, shame, or disgust (e.g., Fiske et al., 2002; Cottrell and Neuberg, 2005; Lickel et al., 2011; Seger et al., 2016) as well as their distinct relationships to behavioral tendencies. Additionally, our measures of episodic and chronic intergroup emotions lacked an explicit reference to respondents’ ingroup membership. Hence, according to Iyer and Leach (2008), we cannot be sure whether we measured intergroup emotions or personal emotions directed at the outgroup. However, several other authors have used comparable items to capture intergroup emotions (e.g., Fernando et al., 2014; Kenworthy et al., 2015). Also, within the context of the original surveys, that mainly dealt with questions related to intergroup relations, it can be assumed that respondents’ ingroup identity was salient. Finally, our ability to generalize the results of these studies is limited to the extent that both studies are situated in the same intergroup context, that is they focus on attitudes and emotions toward and contact with foreigners among German ethnic majority members. Future research should try to replicate our findings in different intergroup contexts. These limitations, however, should be balanced by the strengths of the present paper, including its theoretical novelty, the large heterogenous samples, and the high external validity of these research findings: Our research extends the evidence for the important role of emotions in intergroup relations by using two probability samples. Most previous studies relied on small student samples or other samples of convenience, which pose a threat to the generalization of the findings.

To conclude, we have underlined the importance of considering different types of intergroup emotions for understanding effects of intergroup contact experiences on behavior directed at outgroup members. By doing so, we hope to stimulate further research addressing specific affective and behavioral consequences of positive and negative intergroup contact.

## Author Contributions

OC, MK, FA, UW, TFP, MH, and SJS developed the study concept and drafted the manuscript. MK and OC performed the data analysis and interpretation.

## Conflict of Interest Statement

The authors declare that the research was conducted in the absence of any commercial or financial relationships that could be construed as a potential conflict of interest.

## Footnotes

1 Data for both studies stem from two independent surveys originating from the group-focused enmity project conducted at the University of Bielefeld, Germany (Heitmeyer, 2005, 2006; see also Zick et al., 2008). Surveys contained, among other things, measures of prejudice toward different minority groups, national identification, relative deprivation, and intergroup contact. Unfortunately, neither of the data sets included all the measures in our model: intergroup contact, episodic and chronic intergroup emotions, and discriminatory intentions. Therefore, we had to test different parts of the model in different studies. A selection of publications building on the project’s data can be found online at http://www.uni-bielefeld.de/ikg/projekte/GMF_Survey.html. None of these publications has addressed relationships between different types of intergroup emotions and intergroup contact. Data files of both surveys are available online via https://dbk.gesis.org/dbksearch/GDesc2.asp?list=\&search=\&search2=\&field=\&field2= \&jahr=\&operator=\&bool=\&bool2=\&maxRec=\&sort=\&DB=e\&no=0034.

2 In both studies, respondents were randomly contacted on the district level. A district is a state organizational unit usually composed of a big city or a number of smaller cities, towns or rural areas. Sizes of districts vary between 35,700 and 3,382,200 inhabitants. Altogether, Germany is divided into 440 districts. Because of this sampling procedure, dependency within the districts cannot be ruled out. Therefore, the ‘complex’ procedure was used, which takes data dependency into account by correcting chi-squares and standard errors of the estimates.

3 In the five-factor model, all items had standardized loadings on the respective latent variables between β = 0.614 and β = 0.833.

4 In the five-factor model, all items had standardized loadings on the respective latent variables between β = 0.558 and β = 0.911.

5 We also tested a (statistically equivalent) model in which the order of predictors (episodic emotions) and mediators (chronic emotions) was reversed. In this model a significant indirect effect of chronic anger on aggression via episodic anger emerged (*b* = 0.05, *SE* = 0.024, *p* = 0.024, *CI_95%_* = 0.007, 0.100) but there was no indirect effect of episodic fear on avoidance via episodic fear (*b* = 0.03, *SE* = 0.021, *p* = 0.177, *CI_95%_* = -0.013, 0.071). These results provide additional support for our theoretical model.

## References

[B1] Aberson C. L. (2015). Positive intergroup contact, negative intergroup contact, and threat as predictors of cognitive and affective dimensions of prejudice. *Group Process. Intergroup Relat.* 18 743–760. 10.1177/1368430214556699

[B2] Aberson C. L. Haag S. C. (2007). Contact, perspective taking, and anxiety as predictors of stereotype endorsement, explicit attitudes, and implicit attitudes. *Group Process. Intergroup Relat.* 10 179–201. 10.1177/1368430207074726

[B3] Aberson C. L. McVean A. D. W. (2008). Contact and anxiety as predictors of bias toward the homeless. *J. Appl. Soc. Psychol.* 38 3009–3035. 10.1111/j.1559-1816.2008.00423.x

[B4] Allport G. W. (1954). *The Nature of Prejudice.* Reading, MA: Addison-Wesley.

[B5] Asbrock F. Lemmer G. Becker J. Koller J. Wagner U. (2014). “Who are these foreigners anyway?” – The content of the term foreigner and its impact on prejudice. *Sage Open* 4 1–8. 10.1177/2158244014532819

[B6] Barlow F. K. Paolini S. Pedersen A. Hornsey M. J. Radke H. R. Harwood J. (2012). The contact caveat negative contact predicts increased prejudice more than positive contact predicts reduced prejudice. *Pers. Soc. Psychol. Bull.* 38 1629–1643. 10.1177/0146167212457953 22941796

[B7] Brewer M. B. Miller N. (1984). “Beyond the contact hypothesis: theoretical perspectives on desegregation,” in *Groups in Contact: The Psychology of Desegregation*, eds Miller N. Brewer M. B. (New York, NY: Academic Press), 281–302.

[B8] Cottrell C. A. Neuberg S. L. (2005). Different emotional reactions to different groups: a sociofunctional threat-based approach to “prejudice”. *J. Pers. Soc. Psychol.* 88 770–789. 10.1037/0022-3514.88.5.770 15898874

[B9] Cuddy A. J. Fiske S. T. Glick P. (2007). The BIAS map: behaviors from intergroup affect and stereotypes. *J. Pers. Soc. Psychol.* 92 631–648. 10.1037/0022-3514.92.4.631 17469949

[B10] Dixon J. Durrheim K. Tredoux C. (2005). Beyond the optimal contact strategy: a reality check for the contact hypothesis. *Am. Psychol.* 60 697–711. 10.1037/0003-066X.60.7.697 16221003

[B11] Federal Agency for Civic Education (2015). *Die soziale Situation in Deutschland [The Social Situation in Germany].* Available at: http://www.bpb.de/nachschlagen/zahlen-und-fakten/soziale-situation-in-deutschland/61646/migrationshintergrund-i

[B12] Fernando J. W. Kashima Y. Laham S. M. (2014). Multiple emotions: a person-centered approach to the relationship between intergroup emotion and action orientation. *Emotion* 14 722–732. 10.1037/a0036103 24749637

[B13] Fiske S. T. Cuddy A. J. C. Glick P. Xu J. (2002). A model of (often mixed) stereotype content: competence and warmth respectively follow from perceived status and competition. *J. Pers. Soc. Psychol.* 82 878–902. 10.1037/0022-3514.82.6.878 12051578

[B14] Gaertner S. L. Dovidio J. F. (2000). *Reducing Intergroup Bias: The Common Ingroup Identity Model.* London: Taylor & Francis.

[B15] Graf S. Paolini S. Rubin M. (2014). Negative intergroup contact is more influential, but positive intergroup contact is more common: assessing contact prominence and contact prevalence in five Central European countries. *Eur. J. Soc. Psychol.* 44 536–547. 10.1002/ejsp.2052

[B16] Hayward L. E. Tropp L. R. Hornsey M. J. Barlow K. B. (2017). Toward a comprehensive understanding of intergroup contact: descriptions and mediators of positive and negative contact among majority and minority groups. *Pers. Soc. Psychol. Bull.* 43 347–364. 10.1177/0146167216685291 28903695

[B17] Heitmeyer W. (2005). “Gruppenbezogene Menschenfeindlichkeit. Die theoretische Konzeption und empirische Ergebnisse aus den Jahren 2002 2003 und 2004” in *Deutsche Zustände Folge 3*, ed. Heitmeyer W. (Frankfurt: Suhrkamp), 13–36.

[B18] Heitmeyer W. (2006). “Gruppenbezogene Menschenfeindlichkeit. Gesellschaftliche Zustände und Reaktionen in der Bevölkerung 2002 bis 2005” in *Deutsche Zustände Folge 4*, ed. Heitmeyer W. (Frankfurt: Suhrkamp), 16–37.

[B19] Hewstone M. (1994). “Revision and change of stereotypic beliefs: in search of the elusive subtyping model,” in *European Review of Social Psychology* Vol. 5 eds Stroebe W. Hewstone M. (Chichester: Wiley), 69–109.

[B20] Hewstone M. (2009). Living apart, living together? The role of intergroup contact in social integration. *Proc. Br. Acad.* 162 243–300. 10.5871/bacad/9780197264584.003.0009

[B21] Iyer A. Leach C. W. (2008). Emotion in inter-group relations. *Eur. Rev. Soc. Psychol.* 19 86–125. 10.1080/10463280802079738

[B22] Kenworthy J. B. Voci A. Al Ramiah A. Tausch N. Hughes J. Hewstone M. (2015). Building trust in a postconflict society: an integrative model of cross-group friendship and intergroup emotions. *J. Confl. Resolut.* 60 1041–1070. 10.1177/0022002714564427

[B23] Kessler T. Hollbach S. (2005). Group-based emotions as determinants of ingroup identification. *J. Exp. Soc. Psychol.* 41 677–685. 10.1016/j.jesp.2005.01.001

[B24] Lickel B. Steele R. R. Schmader T. (2011). Group-based shame and guilt: emerging directions in research. *Soc. Pers. Psychol. Compass* 5 153–163. 10.1111/j.1751-9004.2010.00340.x

[B25] Mackie D. M. Devos T. Smith E. R. (2000). Intergroup emotions: explaining offensive action tendencies in an intergroup context. *J. Pers. Soc. Psychol.* 79 602–616. 10.10371/0022-3514.79.4.602 11045741

[B26] Mackie D. M. Maitner A. T. Smith E. R. (2009). “Intergroup emotions theory,” in *Handbook of Prejudice, Stereotyping and, Discrimination*, ed. Nelson T. D. (New York, NY: Psychology Press), 285–307.

[B27] Mackie D. M. Smith E. R. (2015). “Intergroup emotions,” in *Handbook of Personality and Social Psychology*, eds Mikulincer M. Shaver P. R. (Washington, DC: American Psychological Association), 263–293.

[B28] Mazziotta A. Rohmann A. Wright S. C. De Tezanos-Pinto P. Lutterbach S. (2015). (How) Does positive and negative extended cross-group contact predict direct cross-group contact and intergroup attitudes? *Eur. J. Soc. Psychol.* 45 653–667. 10.1002/ejsp.2110

[B29] Miller D. A. Smith E. R. Mackie D. M. (2004). Effects of intergroup contact and political predispositions on prejudice: role of intergroup emotions. *Group Process. Intergroup Relat.* 7 221–237. 10.1177/1368430204046109

[B30] Muthén B. O. Satorra A. (1995). Complex sample data in structural equation modeling. *Sociol. Methodol.* 25 267–316. 10.2307/271070

[B31] Muthén L. K. Muthén B. O. (1998–2015). *Mplus User’s Guide*, 6th Edn. Los Angeles, CA: Muthén & Muthén.

[B32] Paolini S. Harris N. C. Griffin A. S. (2015). Learning anxiety in interactions with the outgroup: towards a learning model of anxiety and stress in intergroup contact. *Group Process. Intergroup Relat.* 19 1–39. 10.1177/1368430215572265

[B33] Paolini S. Hewstone M. Voci A. Harwood J. Cairns E. (2006). “Intergroup contact and the promotion of intergroup harmony: the influence of intergroup emotions,” in *Social Identities: Motivational, Emotional, and Cultural Influences*, eds Brown R. Capozza D. (Hove: Psychology Press), 209–238.

[B34] Parkinson B. Fischer A. H. Manstead A. S. R. (2005). *Emotion in Social Relations.* New York, NY: Psychology Press.

[B35] Pettigrew T. F. Hewstone M. (2017). The single factor fallacy: implications of missing critical variables from an analysis of intergroup contact theory. *Soc. Issues Policy Rev.* 11 8–37. 10.1111/sipr.12026

[B36] Pettigrew T. F. Tropp L. R. (2006). A meta-analytic test of intergroup contact theory. *J. Pers. Soc. Psychol.* 90 751–783. 10.1037/0022-3514.90.5.751 16737372

[B37] Pettigrew T. F. Tropp L. R. (2008). How does intergroup contact reduce prejudice? Meta-analytic tests of three mediators. *Eur. J. Soc. Psychol.* 38 922–934. 10.1002/ejsp.504

[B38] Pettigrew T. F. Tropp L. R. (2011). *When Groups Meet: The Dynamics of Intergroup Contact.* Philadelphia, PA: Psychology Press.

[B39] Reimer N. K. Becker J. C. Benz A. Christ O. Dhont K. Klocke U. (2017). Intergroup contact and social change: implications of negative and positive contact for collective action in advantaged and disadvantaged groups. *Pers. Soc. Psychol. Bull.* 43 121–136. 10.1177/014616721667647 28903647

[B40] Satorra A. Bentler P. M. (2001). A scaled difference chi-square test statistic for moment structure analysis. *Psychometrika* 66 507–514. 10.1007/BF02296192 PMC2905175 20640194

[B41] Schafer J. L. Graham J. W. (2002). Missing data: our view of the state of the art. *Psychol. Methods* 7 147–177. 10.1037//1082-989X.7.2.147 12090408

[B42] Seger C. R. Banerji I. Hee Park S. Smith E. R. Mackie D. M. (2016). Specific emotions as mediators of the effect of intergroup contact on prejudice: findings across multiple participant and target groups. *Cogn. Emot.* 31 923–936. 10.1080/02699931.2016.1182893 27206543

[B43] Smith E. R. (1993). “Social identity and social emotions: toward new conceptualizations of prejudice,” in *Affect, Cognition, and Stereotyping: Interactive Processes in Group Perception*, eds Mackie D. M. Hamilton D. L. (San Diego, CA: Academic Press), 297–315.

[B44] Smith E. R. Mackie D. M. (2005). “Aggression, hatred, and other emotions,” in *On the Nature of Prejudice*, eds Dovidio J. F. Glick P. Rudman L. A. (Malden, MA: Blackwell), 361–376.

[B45] Stangor C. Sullivan L. A. Ford T. E. (1991). Affective and cognitive determinants of prejudice. *Soc. Cogn.* 9 359–380. 10.1521/soco.1991.9.4.359

[B46] Swart H. Hewstone M. Christ O. Voci A. (2011). Affective mediators of intergroup contact: a three-wave longitudinal study in South Africa. *J. Pers. Soc. Psychol.* 101 1221–1238. 10.1037/a0024450 21728450

[B47] Talaska C. A. Fiske S. T. Chaiken S. (2008). Legitimating racial discrimination: emotions, not beliefs, best predict discrimination in a meta-analysis. *Soc. Justice Res.* 21 263–396. 10.1007/s11211-008-0071-2 24052687 PMC3775550

[B48] Tausch N. Hewstone M. Roy R. (2009). The relationships between contact, status and prejudice: an integrated threat theory analysis of Hindu–Muslim relations in India. *J. Community Appl. Soc. Psychol.* 19 83–94. 10.1002/casp.984

[B49] Techakesari P. Barlow F. K. Hornsey M. J. Sung B. Thai M. Chak J. L. (2015). An investigation of positive and negative contact as predictors of intergroup attitudes in the United States, Hong Kong, and Thailand. *J. Cross Cult. Psychol.* 46 454–468. 10.1177/0022022115570313

[B50] Tropp L. R. Pettigrew T. F. (2005). Differential relationships between intergroup contact and affective and cognitive dimensions of prejudice. *Pers. Soc. Psychol. Bull.* 31 1145–1158. 10.1177/0146167205274854 16000274

[B51] van Zomeren M. Spears R. Leach C. W. (2008). Exploring psychological mechanisms of collective action: does relevance of group identity influence how people cope with collective disadvantage? *Br. J. Soc. Psychol.* 47 353–372. 10.1348/014466607X231091 17697447

[B52] Visintin E. P. Green E. G. Pereira A. Miteva P. (2017). How positive and negative contact relate to attitudes towards Roma: comparing majority and high-status minority perspectives. *J. Community Appl. Soc. Psychol.* 27 240–252. 10.1002/casp.2309

[B53] Wagner U. Christ O. (2007). “Intergroup aggression and emotions: a framework and first data,” in *Emotions and Aggressive Behavior*, eds Gollwitzer M. Steffgen G. (Göttingen: Hogrefe & Huber), 133–148.

[B54] Wolsko C. Park B. Judd C. M. Bachelor J. (2003). Intergroup contact: evaluations and perceived variability. *Group Process. Intergroup Relat.* 6 93–110. 10.1177/1368430203006001014

[B55] Zanna M. P. Rempel J. K. (1988). “Attitudes: a new look at an old concept,” in *The Social Psychology of Knowledge*, eds Bar-Tal D. Kruglanski A. (Cambridge: Cambridge University Press), 315–334.

[B56] Zick A. Wolf C. Küpper B. Davidov E. Schmidt P. Heitmeyer W. (2008). The syndrome of group-focused enmity: the interrelation of prejudices tested with multiple cross-sectional and panel data. *J. Soc. Issues* 64 363–383. 10.1111/j.1540-4560.2008.00566.x

